# Radiation Biomarkers: Silver Bullet, or Wild Goose Chase?

**DOI:** 10.3390/jpm11070603

**Published:** 2021-06-25

**Authors:** Eric Andreas Rutten, Christophe Badie

**Affiliations:** 1 Cancer Mechanisms and Biomarkers Group, Radiation Effects Department, Centre for Radiation, Chemical & Environmental Hazards Public Health England Chilton, Didcot OX11 0RQ, UK; Christophe.badie@phe.gov.uk; 2 Department of Oncology, University of Oxford, Oxford OX1 4BH, UK

Humans have learned to harness the power of radiation for therapeutic ends, with 50% of all patients diagnosed with cancer undergoing radiotherapy as part of their treatment [1], second only to surgery, with technical progress evident with new machines such as Cyberknife, MR-Linac, proton or carbon ion [2] therapy and the highly promising flash irradiation [3]. Radiotherapy is an important and effective therapy in the arsenal to treat numerous solid tumours. Improving the precision of radiotherapy has long been the quest of cancer scientists and clinicians. Recent improvements in its precision have helped to deliver most of the dose to the tumour, sparing the surrounding healthy tissues, hence limiting radiation toxicity and long-term effects, such as therapy-related cancer caused by, amongst others, a combination of radiation-induced somatic mutations, modifications of the microenvironment, and inflammation.

However, therapeutic radiation can still potentially lead to harmful secondary effects on patients. Humans exhibit much variability, with unique disease pathologies, especially concerning cancers [4]. The need for medicine to treat patients as individuals, rather than a general “one size fits all” method, is needed. Despite recent significant advances in this field of personalized medicine, the 5-year survival rate of certain cancers, such as lung cancers, remains dismally low, without much improvement, due to a dearth of biomarkers for individual radiosensitivity. However, even with precise planning, radiotherapy treatment outcome depends on multiple factors and can, in the process, damage healthy tissue with a degree of severity that is, until now, exceedingly difficult to predict. Moreover, radiotherapy induces cancer cell death by damaging their DNA and can also trigger the release of pro- and anti-inflammatory mediators. As such, biomarkers are needed to inform and guide the clinicians (external beam and molecular radiation therapy) for them to identify ideal treatment, total dose and fractionation regime as well as assess the toxicity and long-term risks on an individual basis.

Biomarkers for cancer fall into two primary categories: diagnostic and predictive. In the case of radiotherapy for cancers, there is a need for both: (1) diagnosis of radiation response and subsequent effect on the cancer and (2) prediction of possible secondary cancers arising in the long term because of radiotherapy, e.g., acute myeloid leukaemia and sarcomas. Radiation biomarkers are thus necessary, not only for understanding the actual effects of treatment on a tumour, but also for monitoring the most effective total dose to the tumour and identifying mechanisms that may allow a tumour to resist radiation therapy, as well as identifying the risks to the patient stemming from the therapy. Ever-evolving technologies should allow the detection and validation of emerging or new radiation biomarkers, whether genetic, epigenetic, cell-based or cell-free.

In terms of personalised medicine, biomarkers are especially important for tailoring cancer treatment purposes. This refers to a twofold issue: First, we are all different. Not two cancers are alike, with a multitude of unique factors amongst them genetic, epigenetic and inflammatory at play within each tumour. To effectively tailor a cancer therapy regimen for a patient, one must ideally know the radiosensitivity of the tumour. Moreover, studies focusing on tumour microenvironment have provided a better understanding of the effects of clinical radiation therapy and how it is influenced by the famous R’s (Repair, Redistribution, Reoxygenation, Repopulation, Radiosensitivity and Reactivation of an anti-tumour response in the case of combination of immunotherapy/radiotherapy. Importantly for radiation protection purposes, the identification of the patient’s individual susceptibility to radiation would be valuable perhaps especially for paediatric radiotherapy.

Biomarkers are essential for predicting and/or monitoring radiation exposure-associated effects. They take on many forms. In this Special Issue alone, multiple different kinds of biomarkers are explored and reviewed, from proteins such as TGF-β to miRNAs, and more classic methods based on cytogenetics or cytogenetic assays. Currently, there is much argument in the field as to the reliability and clinical application of different biomarker methods, which require professional researcher oversight and assessment, meaning that widescale processing of samples, necessary for true implementation of personalised medicine, remains difficult. As such, the push for biomarkers which lend themselves to upscaled processing methods, or even automated assays, is of vital importance. A question though thus remains: are radiation biomarkers a proverbial silver bullet for personalised treatment of cancers and an essential step towards the improvement of treatment? Or is there simply too much variability and biological noise to integrate and ever achieve, with confidence, a biomarker panel allowing for a holistic insight allowing for individualised courses of radiotherapy?

Overall, the aim of this Special Issue is to present an insight into some of the ongoing research in the oncology radiation response biomarker field and its applications in the ever-evolving field of personalised medicine.

Understanding of the relative biological effectiveness (RBE) of new radiotherapy techniques on cancers and biological states is critical, and a field in which new biomarkers of radiosensitivity and biological effect are indispensable. Naoto Osu et al. [5] demonstrated that carbon ion radiotherapy is more effective in HPV-negative head-and-neck squamous cell carcinomas than in HPV-positive carcinomas, indicating that HPV status is an important prognostic factor for patients. Daijiro Kobayashi et al. [6], in turn, have shown that micronuclei can be detected in solid tumours treated by radiotherapy; they are most likely signalling the activation of antitumour immune responses, showing that micronuclei formation could be an important indicator of radiotherapy effectivity. Niall M. Byrne et al. [7] discussed mechanisms of radioresistance in the tumour microenvironment, including predictive biomarkers for tumour fate post-radiotherapy. Studies such as these demonstrate the vital interplay between radiotherapy and biomarkers, whether they are biomarkers of RBE, or biomarkers demonstrating the opposite in the form of radioresistance within the tumour.

As shown by Mariola Śliwińska-Mossoń et al. [8] in their review, despite advances in understanding the molecular biology of lung cancer and the development of new therapeutic agents, the 5-year overall survival rate of non-small cell lung carcinoma (NSCLC) patients has remained largely the same for decades, at sub-16%. This is a powerful demonstrator of how, despite significant advances in the science of understanding NSCLC and how to treat it, general treatment schedules can only go so far and a deeper look into individuals is required. The review indicates that pre-clinical data supports the idea of miRNA being one such factor to consider.

Interestingly, Jared Luxton et al. [9] proposed to use telomere length and chromosomal instability to predict individual radiosensitivity, using a machine learning model trained on clinical data to predict post-therapy outcomes. Their method shows incredible promise: based on mean telomere length (MTL), the type of long-term secondary effect can be distinguished. Lower MTLs post radiation indicates a propensity for degenerative radiation disease, while higher MTLs demonstrate a predilection towards proliferative cancers. However, the authors themselves are quick to point out that while their machine learning model was effective under their conditions, there is no guarantee that it would also yield comparable results when used on data sets derived under different clinical parameters. Nevertheless, especially given the development of machine learning models and their ever-increasing computational power, this is but a temporary hurdle.

Tools for developing and assessing the viability of such biomarkers panels are already being developed, with one such example within this Special Issue. In their protocol, Anne Dietz et al. [10] outline a procedure by which the effects of ionizing radiation on the human proteome can be determined, with a focus on radiosensitivity. Their method allows for a comprehensive insight into biomarker databases and the generation of a confidence rating, allowing for the development of a panel pooling many different studies and publications, ranked by reliability. Protocols such as this are probably vital for the development of personalized medicine and will doubtless prove highly useful to researchers in the future for biomarker development.

Questions arise as to the source of biomarkers with most promises: what is best? One specific biological sample being sufficient or a combination of them? While messengers such as extracellular vesicles (EVs) seem like promising mines of biomarkers, with Katalin Balázs et al. reporting on a blood-derived panel for prostate cancer utilising EV-miRNA [11], nevertheless doubts can be cast as to their ability to demonstrate radiation-specific markers, as shown in Anna Wojakowska et al. [12], with their pilot study into metabolic profiles of whole serum and serum-derived EVs in head and neck cancer (HNC). While radiation therapy displayed a clear pattern of changes on the serum metabolome, the same could not be said for EVs, which failed to reveal a specific pattern of metabolite changes. However, other sources of research indicate that EVs are a rich source of miRNAs, which have been shown on multiple occasions to have the potential to be powerful indicators of radiation exposure, sensitivity, disease presence and progression.

Furthermore, questions regarding methodology remain, as shown by Takahiro Oike et al. [13] in their paper on clonogenic assays to assess in vitro radiosensitivity, in which they compare whether pre- or post-IR plating has an impact on the outcome, concluding that there is a negligible difference. Simon Sioen et al. [14] continue this vein of standardizing methodology by investigating whether the cytokinesis-block micronucleus assay works on both fresh and cryopreserved PBMCs, ultimately developing a standardized assay useable on both. Work such as this is important in not only establishing a consensus, but also allowing for inter-study comparison.

Despite the optimism transpiring from the findings described above it remains important to realize that the actual application of biomarkers remains limited, and as such they are, as of yet, wishful thinking. Without actual widescale clinical application methods, their impact and relevance to personalized medicine remains theoretical. The limitations of current methods are outlined by Volodymyr Vinnikov et al.’s [15] insightful review on ex vivo cytogenetic radiation biomarkers, and logistical issues dealing with large scale studies are shown in Jayne Moquet et al.’s [16] RENEB report, detailing the problems faced by undertaking a large scale human health effect study; this same logistical issue could be faced when dealing with a large cohort of patients and an individualized care approach to each. Prabal Subedi et al.’s [17] review concludes that while most IR biomarker studies use repair foci as their biomarker of choice, their actual association to final clinical outcomes remains contradictory. However, not all is lost, despite such limitations and issues: both articles contain plans for overcoming such problems, and as such for transforming radiation biomarkers from theoretical marvels into practical, clinical solutions for cancer radiotherapy.

Doubtless, many questions remain regarding the validity and practicality of using a panel of biomarker to drive treatment decision ultimately leading to personalized medicine. However, significant inroads have been made in recent years, elucidating the radiation response of tumours, and biomarkers have been derived therefrom. However, there is undoubtedly much to be discovered, and even more to be optimized—the road from lab result to clinical application is still long and fraught with challenges. As such, one must be forced to conclude that biomarkers are not, in fact, some proverbial silver bullet, at least for now. They are difficult to identify and validate, and, ultimately, possibly too expensive, time consuming or complex to implement. However, biomarkers are not a fool’s errand—far from it. In fact, although the knowledge of radiation biomarkers is currently insufficient for widescale implementation of personalized cancer treatments, it does not mean that this will continue to be the case in the near future. In fact, with the expansion of studies as well as the development of ever more sophisticated technologies, one would hope that reliable and useful radiation biomarkers are on the cusp of becoming a silver bullet. Given the articles published in this Special Issue, it is not unwise to be optimistic. The dawn of radiation biomarkers is approaching, bringing with it a new horizon of therapeutic possibilities.

## Data Availability

Not applicable.
